# The First Case of the Identification of a Microorganism Directly from Whole Blood Using MALDI-TOF Mass Spectrometry in an Onco-Hematological Pediatric Patient with Bloodstream Infection

**DOI:** 10.3390/antibiotics14020149

**Published:** 2025-02-04

**Authors:** Venere Cortazzo, Maria del Carmen Pereyra Boza, Vanessa Tuccio Guarna Assanti, Gianluca Foglietta, Gianluca Vrenna, Marilena Agosta, Elena Chaiter, Martina Rossitto, Barbara Lucignano, Manuela Onori, Valeria Fox, Marco Becilli, Pietro Merli, Filippo Frioni, Carlo Federico Perno, Paola Bernaschi

**Affiliations:** 1 Microbiology and Diagnostic Immunology Unit, Bambino Gesù Children’s Hospital, IRCCS, 00165 Rome, Italy; venere.cortazzo@opbg.net (V.C.); mdcarmen.pereyraboza@opbg.net (M.d.C.P.B.); vanessa.tuccio@opbg.net (V.T.G.A.); marilena.agosta@opbg.net (M.A.); barbara.lucignano@opbg.net (B.L.); manuela.onori@opbg.net (M.O.); carlofederico.perno@opbg.net (C.F.P.); paola.bernaschi@opbg.net (P.B.); 2 Multimodal Laboratory Medicine, Bambino Gesù Children’s Hospital, IRCCS, 00165 Rome, Italy; elena.chaiter@opbg.net (E.C.); martina.rossitto@opbg.net (M.R.); valeria.fox@opbg.net (V.F.); 3 Department of Hematology/Oncology, Cell and Gene Therapy, Bambino Gesù Children’s Hospital, IRCCS, 00165 Rome, Italy; marco.becilli@opbg.net (M.B.); pietro.merli@opbg.net (P.M.); 4 Section of Hematology, Department of Radiological and Hematological Sciences, Catholic University of the Sacred Heart, 00168 Rome, Italy; filippo.ffrioni@outlook.com

**Keywords:** whole blood, MALDI-TOF mass spectrometry, pediatric patient, direct pathogen identification

## Abstract

Background: Bloodstream infections affect up to 20% of pediatric cancer patients receiving intensive care, contributing significantly to morbidity and mortality, with infection-related mortality rates reported to be as high as 16%. Methods: The identification of microorganisms directly from whole blood is difficult due to several factors, such as interference from host genomic material, low bacterial load, the endogenous components of whole blood and exogenous substances, which can interfere with the identification process. Nevertheless, rapid microbial diagnosis remains of paramount importance in these patients. Results and Conclusion: Here, we present the first case of bacterial pathogen identification directly from whole blood using MALDI-TOF mass spectrometry in an onco-hematological pediatric patient affected by sepsis and admitted to Bambino Gesù Children’s Hospital (IRCCS) in Rome, Italy.

## 1. Introduction

Bloodstream infection (BSI) occurs when pathogens enter the bloodstream and spread systemically. In severe cases, BSI can progress to sepsis, leading to shock, disseminated intravascular coagulation and multiple organ failure. The incidence of BSI ranges between 113 and 204 cases per 100,000 people [1,2], and these numbers have been rising annually [3,4]. BSIs are also a significant cause of morbidity and mortality in pediatric cancer patients undergoing intensive treatment regimens [5]. Among children who die of cancer within five years of diagnosis, 75% succumb to disease progression, while 21.4% die from treatment-related causes [6], with infections accounting for approximately 16% of these deaths [7]. These data reflect a significant cause of preventable and avoidable mortality, highlighting the urgency and need for a more rapid approach and targeted preventive strategies. In fact, it is important to note that infections in pediatric cancer patients are driven not only by intensive treatments, but also by the intrinsic immunosuppression associated with the disease.

Several factors increase the vulnerability of pediatric cancer patients to BSIs. Intensive chemotherapy and radiation weaken the immune system, while hematopoietic stem cell transplantation (HSCT) further increases the risk due to its associated immunosuppression, especially in patients with underlying hematologic malignancies [8]. Neutropenia further exacerbates this risk, with sepsis rates reported to be as high as 16% [9,10]. Additionally, most pediatric oncology patients rely on central venous catheters (CVCs), which compromise physical barriers and increase the BSI risk, particularly with external or non-tunneled lines [11]. These factors underscore the critical need for early pathogen detection and timely treatment to improve the clinical outcomes.

Blood culture remains the gold standard for identifying bacteria and fungi in sepsis cases, enabling optimized antimicrobial therapy and treatment efficacy monitoring [12]. However, blood cultures have significant limitations such as prolonged turnaround times, often exceeding 24–48 h without rapid diagnostic tools [13]. Therefore, new diagnostic techniques are needed to overcome these delays.

Matrix-assisted laser desorption/ionization-time-of-flight mass spectrometry (MALDI-TOF MS; Bruker Daltonics, Bremen, Germany) has revolutionized clinical microbiology. Compared to conventional biochemical systems, MALDI-TOF MS significantly reduces diagnostic time by rapidly identifying pathogenic bacteria and fungi, as well as detecting antimicrobial resistance [14]. Initially, MALDI-TOF MS was limited to identifying microorganisms from cultured isolates. However, over time, its applications have expanded to include the direct detection of pathogens from clinical samples, although this approach still requires further exploration. Currently, its use in positive blood cultures for the identification of microorganisms and the detection of antimicrobial resistance represents one of the most useful applications [15].

However, direct pathogen identification from whole blood poses unique challenges. In septic patients, host genomic material can outnumber microbial DNA by up to 10^10^-fold, complicating detection. Additional obstacles include low bacterial loads and interference from hemoglobin, albumin and exogenous substances such as anticoagulants [13]. Despite these difficulties, rapid and precise microbial diagnosis is essential for guiding antimicrobial therapy and preventing sepsis progression. MALDI-TOF MS’s ability to identify pathogens directly from whole blood without prior culture represents a groundbreaking advancement.

This case study describes the first successful use of MALDI-TOF MS to directly identify a bacterial pathogen from whole blood in an onco-hematological pediatric patient with bloodstream infection, enabled by a high bacterial load.

## 2. Case Description

We present the case of a 12-year-old patient diagnosed with T-cell acute lymphoblastic leukemia (T-ALL) admitted to the Hematology Department of Bambino Gesù Children’s Hospital. After receiving a CD7-targeting Chimeric Antigen Receptor (CAR) T-cell infusion, the patient underwent an allogeneic stem cell transplant (HSCT). The post-transplant period was marked by severe complications, including acute renal failure, fungal lung infection, respiratory failure, adenovirus viremia, arterial hypertension and left ventricular failure.

Despite aggressive management, the patient’s condition worsened, ultimately progressing to septic shock. Upon admission to the intensive care unit, blood samples were collected for microbiological analysis, and standard diagnostic protocols, including blood cultures and empirical antibiotic therapy, were initiated.

**Figure 1:**
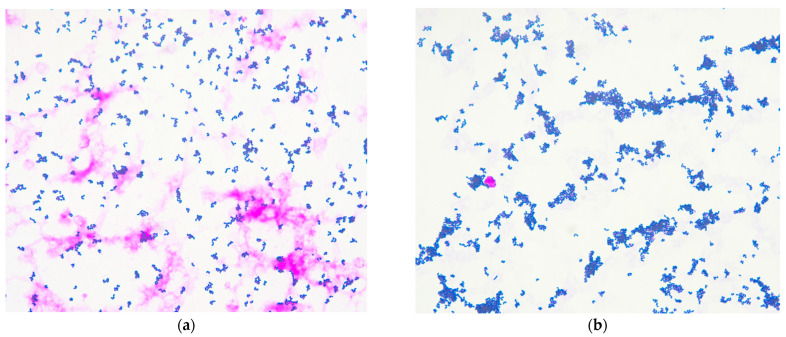
(**a**) Manual smear prepared for peripheral blood platelet count, revealing high bacterial load (original magnification, ×1000). (**b**) Bacterioscopic examination of whole blood sample showing Gram-positive bacilli on Gram staining. Gram-stained bacteria viewed with brightfield microscope at 1000× magnification with oil immersion.

Successive complete blood counts revealed a rapidly increasing platelet count, exceeding 100,000/μL and surpassing 1,000,000/μL within a few hours. A manual platelet count via peripheral blood smear unexpectedly revealed extensive bacterial presence (Figure 1a). This prompted the immediate submission of the whole blood sample to the microbiology laboratory for further investigation and microbiological consultation.

**Figure 2:**
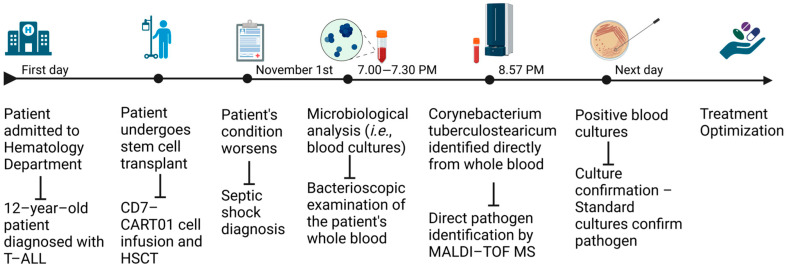
Timeline of microbiological investigations and relevant findings leading to identification of *Corynebacterium tuberculostearicum* by MALDI-TOF MS directly from whole blood. Created in BioRender [16].

Given the patient’s critical condition, rapid pathogen identification was essential to optimize treatment. A timeline of the microbiological investigations and their results is shown in Figure 2.

## 3. Microbiological Investigation and Relevant Findings

A bacterioscopic examination of patient’s whole blood sample revealed a high bacterial load of Gram-positive bacilli (Figure 1b). This unique finding enabled the use of MALDI-TOF MS for direct pathogen identification, bypassing traditional culture-based workflows. Typically, culture-based identification requires 24–48 h from blood collection to pathogen identification.

Therefore, a MALDI-TOF MBT Smart mass spectrometer (Bruker Daltonics, Bremen, Germany) in positive ion mode with a laser frequency of 200 Hz was used for pathogen identification, employing the MALDI Biotyper (Bruker Daltonics, Bremen, Germany) V4.1.14 software package. An instrument calibration was performed with the Bruker Bacterial Test Standard (BTS), and the spectra profiles were analyzed using the Bruker Biotyper V.11.0.0 library alone (covering 3893 species and 10,833 entries) for bacterial identification.

For sample preparation, 1 mL of sample was mixed with 200 µL of lysis buffer from the MALDI Sepsityper kit (Bruker Daltonics, Bremen, Germany) and shaken for 10 s. After centrifugation for 2 min at 13,000 rpm, the supernatant was removed and 500 µL of deionized water was added, and after suspension, the sample was centrifuged again for 1 min at 13,000 rpm. After removing the supernatant, 1 mL of buffer was added, and the pellet was resuspended. The sample was again centrifuged for 1 min at 13,000 rpm and, after removing the buffer, the bacterial pellet was spotted into the target. Seventy percent formic acid was added to each well, and one microliter of α-cyano-4-hydroxycinnamic acid (CHCA) matrix was added to the samples and allowed to air-dry before being placed in the mass spectrometer.

In this case, MALDI-TOF MS directly identified *Corynebacterium tuberculostearicum* from the whole blood, significantly reducing the diagnostic turnaround time and allowing for the rapid initiation of therapy. The direct identification result was provided approximately one hour after the samples arrived at the microbiology laboratory. This is remarkably faster than the time required for the blood cultures to turn positive (mean [SD] = 10.9 [5.5] h), and for final microorganism identification (mean [SD] = 65.7 [24.5] h) [17].

Meanwhile, standard blood cultures were processed using the BacT/ALERT VIRTUO system (bioMérieux, Marcy l’Étoile, France). Blood samples were inoculated into aerobic and anaerobic culture bottles at 6:55 p.m. The aerobic cultures became positive after 3 h and 18 min and the anaerobic cultures after 2 h and 42 min of incubation. Subsequent subcultures on solid media were incubated overnight at 37 °C. The colonies grown on the agar plates were identified the next day using MALDI-TOF MS, which confirmed the identification of *Corynebacterium tuberculostearicum*.

Notably, the microorganism was identified directly from the whole blood before the positive blood cultures, with the MALDI-TOF MS identification report available at 8:57 p.m. (Supplementary Figure S1). This provided a faster identification result, requiring approximately 2 h from the whole blood compared to ~15/18 h for the culture-based identification and ~4/5 h for direct identification from the positive blood cultures. This time-saving (13–16 h or 2–3 h, respectively) can be critical in managing infections, especially in vulnerable patients. These findings highlight the diagnostic advantage of MALDI-TOF MS in high-bacterial load scenarios, where rapid identification can significantly influence clinical decision-making.

Table 1 summarizes the advantages and limitations of using MALDI-TOF MS for direct pathogen identification. The benefits include rapid diagnosis and high accuracy, while the limitations involve the need for high bacterial loads and challenges related to the host genomic material, endogenous whole blood components and exogenous substances. Although some studies suggest a limit of detection of 6 × 10^3^ CFU/spot, a practical limit of 1 × 10^5^ CFU/spot is often necessary [18]. A significant biomass is required to obtain reliable identification results, and these limitations of MALDI TOF MS will need to be addressed in the future.

Taken together, this represents the first reported case of the successful identification of a bacterial pathogen directly from whole blood using MALDI-TOF MS. This result was facilitated by the high bacterial load in the blood, which allowed the detection of the microbial signal despite the presence of endogenous components of the whole blood and exogenous substances that interfered with the identification process.

## 4. Implications and Conclusions

This case highlights the potential of MALDI-TOF MS to revolutionize BSI diagnosis, particularly in critically ill patients with high bacterial loads. By enabling direct pathogen identification from whole blood, this technique bypasses the delays associated with culture-based methods, facilitating earlier, targeted antimicrobial therapy that can be life-saving.

For pediatric oncology patients, a timely and accurate diagnosis is crucial to minimize the use of broad-spectrum antibiotics and reduce the risks of antimicrobial resistance. The success of MALDI-TOF MS in this case highlights its potential in addressing these challenges by delivering rapid and precise pathogen identification. Its ability to detect microorganisms directly from whole blood without prior culture represents a significant shift in clinical microbiology, especially for high-risk populations. However, the application of MALDI TOF MS still remains in early development, with several challenges that still need to be overcome. Standardization is also needed to ensure reliability. Future research should focus on optimizing sample preparation, enhancing the detection of low bacterial loads and expanding pathogen detection libraries.

In conclusion, this study demonstrates the potential of integrating MALDI-TOF MS into routine workflows for direct pathogen identification from whole blood, offering a promising strategy to enhance infection management in vulnerable patient populations. To our knowledge, this is the first reported case of direct bacterial pathogen identification from whole blood using MALDI-TOF MS in a patient with bloodstream infection, marking a significant advancement in rapid diagnostics. MALDI-TOF MS has the potential to become a transformative diagnostic tool in clinical microbiology, particularly for high-risk patients requiring immediate and targeted therapeutic interventions, and whose potentially high bacterial biomasses may make this rapid approach feasible and impactful.

## Figures and Tables

**Table 1:** A summary of the advantages and disadvantages of using MALDI-TOF MS for direct pathogen identification from whole blood.

Advantages	Disadvantages
Rapid diagnosis	High bacterial load required
High accuracy	Presence of host genomic material, endogenous whole blood components and exogenous substances
Direct identification	Limited applicability
Reduced antibiotic exposure	Identification of polymicrobial samples
Improved clinical outcomes	Need for further research

## Data Availability

All data generated or analyzed during this study are included in this published article. Further inquiries can be directed to the corresponding author.

## Supplementary Materials

The following supporting information can be downloaded at: https://www.mdpi.com/article/10.3390/antibiotics14020149/s1, Figure S1: MALDI-TOF MS identification report.
