# Supplementary material for: The First Case of the Identification of a Microorganism Directly from Whole Blood Using MALDI-TOF Mass Spectrometry in an Onco-Hematological Pediatric Patient with Bloodstream Infection
Source: Antibiotics (Basel). 2025 Feb 4;14(2):149. doi: 10.3390/antibiotics14020149 (PMC11851728; doi:10.3390/antibiotics14020149)
Supplement: Supplementary file 1 [file antibiotics-14-00149-s001.zip › antibiotics-3452216-supplementary.pdf]

First case of identification of a microorganism directly from whole blood using MALDI-TOF Mass Spectrometry in an onco-hematological pediatric patient with bloodstream infection

Venere Cortazzo[1†], Maria del Carmen Pereyra Boza[1†], Vanessa Tuccio Guarna Assanti[1†], Gianluca Foglietta[1*], Gianluca Vrenna[2*], Marilena Agosta[1], Elena Chaiter[2], Martina Rossitto[2], Barbara Lucignano[1], Manuela Onori[1], Valeria Fox[2], Marco Becilli[3], Pietro Merli[3], Filippo Frioni[4], Carlo Federico Perno[1] and Paola Bernaschi[1]

[1] Microbiology and Diagnostic Immunology Unit, Bambino Gesù Children’s Hospital, IRCCS, Rome, Italy

[2] Multimodal Laboratory Medicine, Bambino Gesù Children’s Hospital, IRCCS, Rome, Italy

[3] Department of Hematology/Oncology, Cell and Gene Therapy, IRCCS Bambino Gesù Children's Hospital, Rome, Italy

[4] Catholic University of the Sacred Heart, Rome, Italy

Supplementary Figure S1. MALDI-TOF MS identification report of the microorganism directly from the patient's whole blood with the highest score of 2.00. A score of 2.00 represents a score above the threshold for reliable identification according to the manufacturer's guidelines.

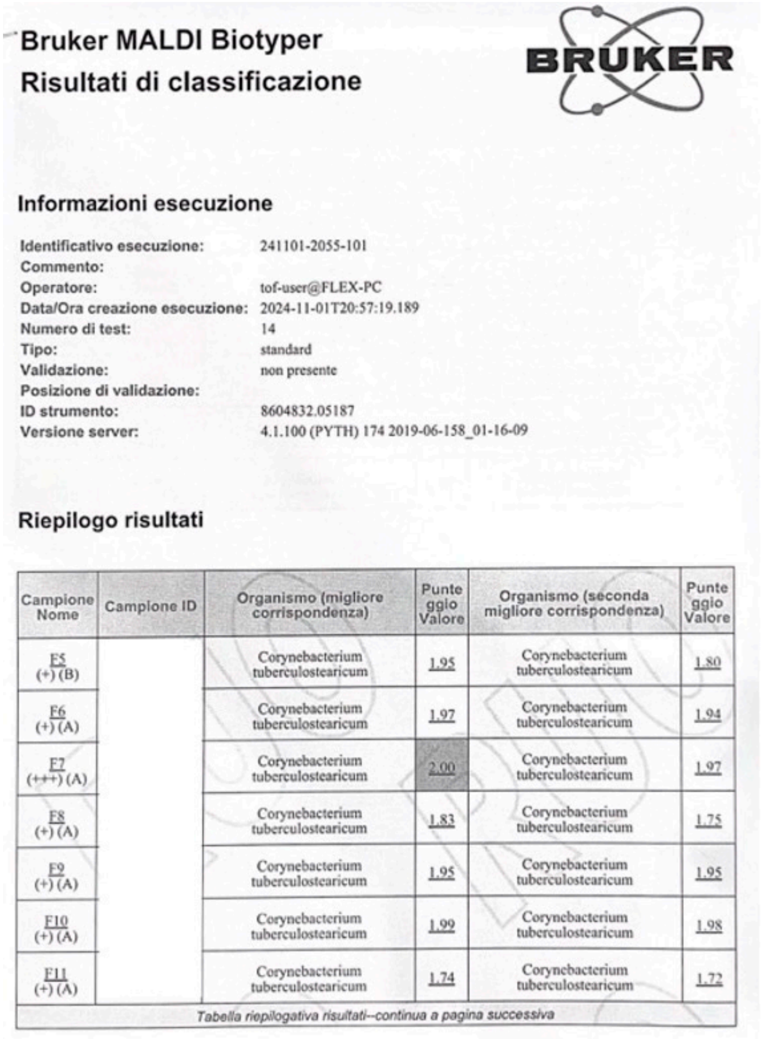

Bruker MALDI Biotyper
Risultati di classificazione

Informazioni esecuzione

Identificativo esecuzione:	241101-2055-101
Commento:	
Operatore:	tof-user@FLEX-PC
Data/Ora creazione esecuzione:	2024-11-01T20:57:19.189
Numero di test:	14
Tipo:	standard
Validazione:	non presente
Posizione di validazione:	
ID strumento:	8604832.05187
Versione server:	4.1.100 (PYTH) 174 2019-06-158_01-16-09

Riepilogo risultati

Campione Nome	Campione ID	Organismo (migliore corrispondenza)	Punteggio Valore	Organismo (seconda migliore corrispondenza)	Punteggio Valore
F5 (+) (B)		Corynebacterium tuberculostearicum	1.95	Corynebacterium tuberculostearicum	1.80
F6 (+) (A)		Corynebacterium tuberculostearicum	1.97	Corynebacterium tuberculostearicum	1.94
F7 (+++) (A)		Corynebacterium tuberculostearicum	2.00	Corynebacterium tuberculostearicum	1.97
F8 (+) (A)		Corynebacterium tuberculostearicum	1.83	Corynebacterium tuberculostearicum	1.75
F9 (+) (A)		Corynebacterium tuberculostearicum	1.95	Corynebacterium tuberculostearicum	1.95
F10 (+) (A)		Corynebacterium tuberculostearicum	1.99	Corynebacterium tuberculostearicum	1.98
F11 (+) (A)		Corynebacterium tuberculostearicum	1.74	Corynebacterium tuberculostearicum	1.72

Tabella riepilogativa risultati--continua a pagina successiva
